# Diagnostic Stability of Primary Psychotic Disorders in a Research Sample

**DOI:** 10.3389/fpsyt.2021.734272

**Published:** 2021-10-28

**Authors:** Andrea J. Wood, Amber R. Carroll, Ann K. Shinn, Dost Ongur, Kathryn E. Lewandowski

**Affiliations:** ^1^Schizophrenia and Bipolar Disorder Research Program, McLean Hospital, Belmont, MA, United States; ^2^Department of Psychiatry, Harvard Medical School, Boston, MA, United States

**Keywords:** psychosis, diagnostic stability, schizoaffective disorder, bipolar disorder, schizophrenia

## Abstract

Psychiatric diagnosis is often treated as a stable construct both clinically and in research; however, some evidence suggests that diagnostic change may be common, which may impact research validity and clinical care. In the present study we examined diagnostic stability in individuals with psychosis over time. Participants with a diagnosis of any psychotic disorder (*n* = 142) were assessed at two timepoints using the Structured Clinical Interview for Diagnostic and Statistical Manual of Mental Disorders. We found a 25.4% diagnostic change rate across the total sample. People with an initial diagnosis of psychosis not otherwise specified and schizophreniform disorder had the highest rates of change, followed by those with schizophrenia and schizoaffective disorder; people with bipolar disorder had the lowest change rate. Most participants with an unstable initial diagnosis of schizophrenia, schizophreniform disorder, bipolar disorder, or psychosis not otherwise specified converted to a final diagnosis of schizoaffective disorder. Participants with an unstable initial diagnosis of schizoaffective disorder most frequently converted to a diagnosis of schizophrenia. Our findings suggest that diagnostic change is relatively common, occurring in approximately a quarter of patients. People with an initial diagnosis of schizophrenia-spectrum disorder were more likely to have a diagnostic change, suggesting a natural stability of some diagnoses more so than others.

## Introduction

Both in research and clinical settings psychiatric diagnosis is often considered a stable construct. However, some evidence suggests that diagnostic change may be common, especially in psychotic disorders such as bipolar disorder (BP), brief psychotic disorder, major depressive disorder with psychotic features (MDD), psychosis not otherwise specified (Psych NOS), schizoaffective disorder (SZA), schizophrenia (SZ), and schizophreniform disorder (SZform).

Substantial debate regarding the utility of categorical vs. dimensional classification in psychotic disorders continues across the psychosis spectrum and in disorders such as SZA in particular (1–6). The Kraepelinian dichotomy draws a sharp boundary between BP and SZ; diagnoses that share characteristics of both disorders may be conceptualized as categorically separate disorders or may be considered to fall along a continuum in which someone may move toward one end or the other reflecting shifting symptom profiles (2, 3). Nevertheless, clear evidence of the superiority of categorical vs. dimensional classification systems has not been demonstrated (7), and pre-defined categorization of diagnoses is commonly used in both clinical and research settings.

Determining the most accurate diagnosis, as indicated by our current construct of illness, early in the trajectory of a patient's illness is relevant to determining best treatment practices and for psychoeducation and communication about the nature and course of illness with the patient and their loved ones (8). While many treatments for psychosis may be similar transdiagnostically, others may not. For example, some psychotropic medications are more effective in some diagnostic groups than others, demonstrating a clear need for accurate diagnosis (9). Better understanding of the frequency and predictors of diagnostic change thus has clinical practice implications.

Diagnostic instability has implications for clinical research as well. Diagnosis based on the Diagnostic and Statistical Manual of Mental Disorders (DSM) is a common grouping variable in psychiatric research and is frequently included among inclusion and exclusion criteria for subject participation (10). While transdiagnostic frameworks that focus on symptom dimensions rather than diagnostic categories as recommended by the National Institute of Mental Health (NIMH) Research Domain Criteria (RDoC) circumvent this issue, DSM or International Classification of Diseases (ICD) defined diagnostic categories are still relevant as they continue to reflect our current classification system. As such, understanding both the prevalence and predictors of diagnostic instability is critically important to conducting effective and accurate research.

Variable stability rates have been reported for psychotic disorders in the literature: meta-analyses have found kappa values ranging from 0.27 to 0.66 for various psychotic disorders (11) and studies report stability rates ranging from 0% to 96.5% depending on sample type (i.e., a research or clinical sample), sample size, and diagnosis (12–16). In people initially diagnosed with SZ, stability rates have ranged from 60 to 82% (12–14, 16), suggesting that at least around one fifth of patients experience a diagnostic change. Stability rates for SZA vary in the extant literature from as low as 0% (13) to 89% (12). BP diagnoses tend to be more stable than SZA diagnoses (17), but this is not consistent across studies. Chen and colleagues reported a stability rate of 71.1% (18), whereas Salvatore and colleagues found 96.5% stability in their sample (2009). However, in a study of people with BP with psychosis over 60% received an initial diagnosis other than BP (19). As might be expected, Psych NOS proves particularly unstable across studies, with stability rates ranging from as low as 13–36% (12, 13).

Both demographic and clinical variables have been associated with diagnostic instability. Younger age at onset and male sex have both been associated with greater diagnostic instability (14, 20), as has the presence of comorbid disorders at initial diagnosis, including comorbid substance use disorders (SUDs) and non-psychiatric medical illnesses. In terms of substance use, Chen and colleagues (18) found that patients with a current or previous diagnosis of a SUD or alcohol use disorder had a higher rate of diagnostic change away from BP, but a lower rate of change away from SZ. Tohen and colleagues (20) reported that lower prevalence of comorbid physical or medical illnesses at initial diagnosis was independently and significantly associated with diagnostic instability. Other factors associated with diagnostic instability include duration of follow-up. One study found that duration of follow-up was longer for people with unstable diagnoses than those with stable diagnoses (21) and another found that longer follow-up duration was more strongly associated with a final diagnosis of SZA compared to BP or MDD (22). These findings may reflect increasing likelihood of alterations in clinical presentation or changes in diagnostic criteria over longer timeframes.

In the present study we aimed to examine diagnostic stability in people with psychosis. Based on evidence from the literature we hypothesized that (1) across the total sample, diagnostic instability would be reported in approximately a quarter of the participants, but would vary by diagnosis, with instability highest in PsychNOS and lowest in BP; (2) diagnostic change from BP to SZA would be more common than the reverse; (3) people with comorbid SUDs would have a higher rate of diagnostic instability; (4) sex and age at onset would be predictive of diagnostic change across the sample, with males and those with younger age at onset showing higher diagnostic instability.

## Methods

### Participants

Participants included 142 research subjects from McLean Hospital's Schizophrenia and Bipolar Disorder Program (SBDP) who had at least two diagnostic assessments using the Structured Clinical Interview for DSM (SCID)-IV-TR or SCID-5 as part of their participation in a research study in the SBDP. All participants were enrolled between 2006 and 2021. People with initial diagnoses of SZ, BP, SZA, SZform, Psych NOS, and MDD were included. Inclusion and exclusion criteria varied by study; however, in all cases participants were between the ages of 18 and 60, had no history of head injury with loss of consciousness, seizure disorder, or mental retardation. The research protocols included in this project were approved by the McLean Hospital Institutional Review Board. All participants provided written informed consent after receiving a complete description of the study.

### Materials

Diagnosis was determined by trained clinical raters using the SCID-IV-TR or the SCID-5. All participants completed mood, psychosis, and differential diagnosis modules. Anxiety and substance use modules were completed for most participants; additional modules were completed inconsistently across studies and therefore were not included in these analyses. Clinical symptoms were assessed using the Young Mania Rating Scale (YMRS) to assess manic symptoms, Montgomery-Asberg Depression Rating Scale (MADRS) to assess depressive symptoms, and Positive and Negative Syndrome Scale (PANSS) to assess psychotic symptoms.

### Procedures

Diagnostic interviews were administered in the context of several separate but related studies, as noted above. SCID interviews were repeated if a participant's previous SCID had been administered more than 6 months prior. Participants received either SCID-IV-TR or SCID-5, depending on the research study initially enrolled in. Some participants received both versions of the SCID at different time points. Interviews were conducted in a single session by trained clinical research staff; interrater reliability for overall diagnosis was very high (23).

### Statistical Analysis

All analyses were conducted in IBM SPSS Statistics software v.28. Diagnostic stability was determined by comparing all participants' diagnoses at initial and final SCIDs. Conversion rates were calculated based on rates of diagnostic change. Participants were grouped based on whether or not their diagnosis changed from initial to final assessment; “non-converters” and “converters” were then compared on demographic and clinical measures at initial assessment using Chi-squared or *t*-test, as appropriate. Logistic regressions were then performed to examine whether diagnosis at initial assessment and variables have been found to be associated with diagnostic instability including sex, age at onset, and SUD predicted change in diagnosis. For the logistic regressions, diagnostic groups were combined into two broad categories: mood disorders (including BP and MDD) and SZ Spectrum (including SZ, SZA, SZform, and PsychNOS) in order to reduce the levels of diagnosis as a predictor for better interpretability, and because several of the diagnostic categories contained very few participants. Statistical significance for all analyses was set at *p*< 0.05.

## Results

### Demographic and Clinical Variables

**Figure 1:**
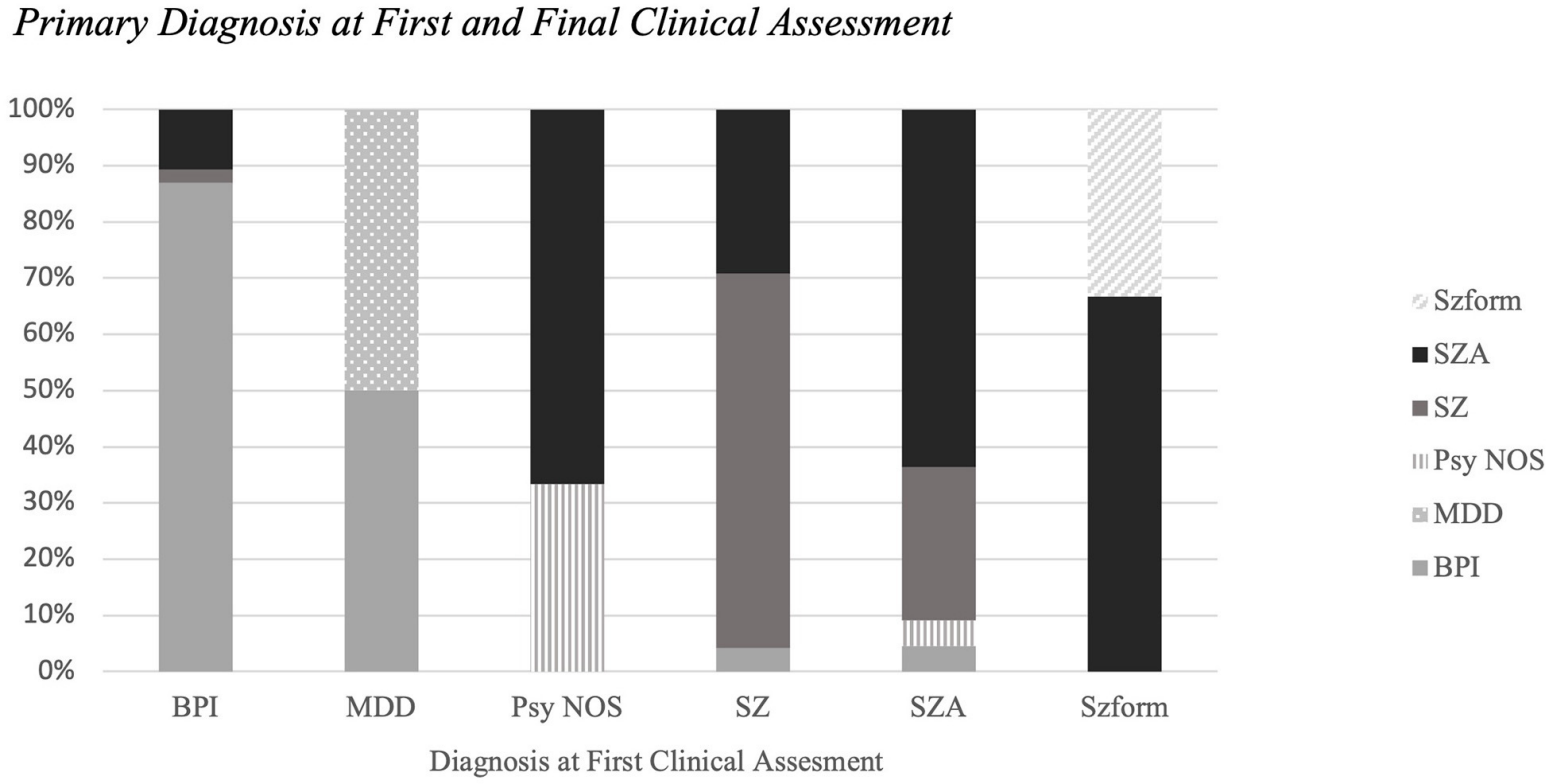
Primary diagnosis at first and final clinical assessment. The distribution of primary psychotic disorder diagnoses at first clinical assessment and diagnoses at final clinical assessment. Diagnostic stability rates differed by psychotic disorders; in order from greatest to least: BP (86.9%), SZ (66.7%), SZA (63.6%), Psych NOS (33.3%), SZform (33.3%).

There were 339 diagnostic assessments across all 142 subjects (75.8% SCID-IV-TR). Mean number of assessments per subject was 2.38 (SD = 0.98, median 2, range 2–8) and mean duration between the initial and final assessments was 543.3 days (range 2.0–4575.0 days, median 236 days, SD = 730.0 days). The distribution of primary psychotic disorder diagnoses at first clinical assessment from most to least frequent was as follows: BP, SZ, SZA, SZform, Psych NOS, and MDD. The distribution of frequencies at the final clinical assessment shifted slightly: BP, SZA, SZ, Psych NOS, SZform, and MDD (Figure 1).

**Figure 2:**
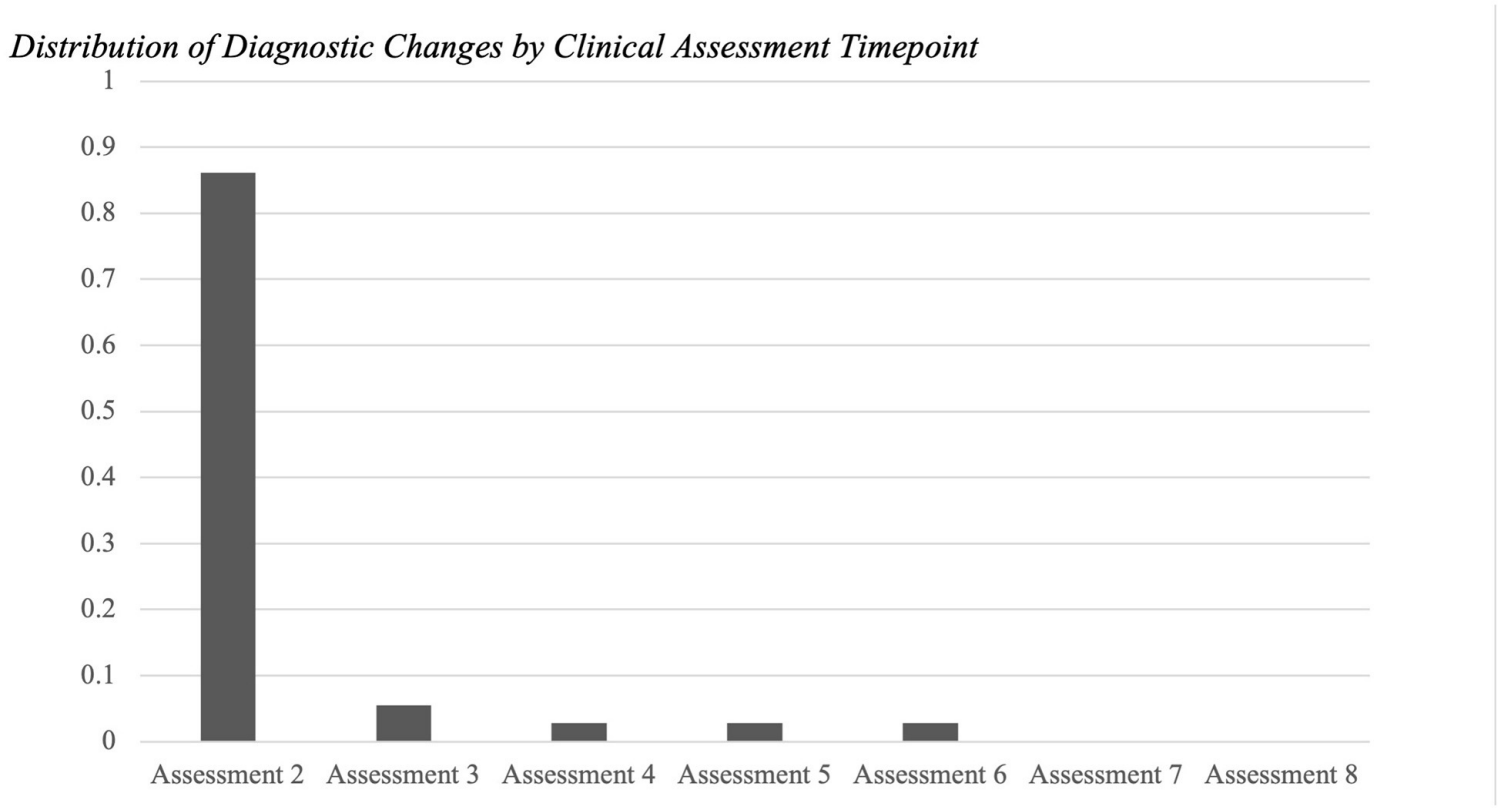
Distribution of diagnostic changes by clinical assessment timepoint. Out of the 36 diagnostic changes that were recorded, 86% occurred at the time of the second clinical assessment (*N* = 142). 6% of the changes occurred at the third time point (*N* = 27). Followed by fourth (*N* = 15), fifth (*N* = 7), and sixth (*N* = 3) (all 3%). No diagnostic changes occurred at the seventh or eighth timepoints for any participant (*N* = 2 and *N* = l, respectively).

In the subset of participants with diagnostic changes, most people with an initial diagnosis of SZ (87.5%), schizophreniform (66.7%), BP (81.8%), or Psych NOS (100%) converted to a final diagnosis of SZA. Participants with an unstable initial diagnosis of SZA were most likely to convert to a final diagnosis of SZ (75.0%). Out of the 36 diagnostic changes that were recorded, 86% occurred at the time of the second clinical assessment (Figure 2).

Comparison of converters and non-converters showed that converters had significantly earlier age at onset and significantly lower rates of SUD (Table 1). SCID type at first clinical assessment was associated with a later diagnostic change, with more SCID-IV-TR assessments associated with a switch than SCID-5-RV (*p* = 0.036). Twenty two percent (22.2%) of subjects with an unstable diagnosis initially had a SCID-IV-TR and then SCID-5-RV; however, inconsistent SCID type was not significantly associated with a change in diagnosis χ^2^ (1, *N* = 142) = 0.006, *p* = 0.94. Unexpectedly, the non-converter group had a higher proportion of comorbid SUD than converters; χ^2^ (1, *N* = 142) = 6.375, *p* = 0.012. Groups did not differ by rate of comorbid anxiety disorders (*p* = 0.207), and there were no other significant groups differences on any demographic or clinical measures.

**Table 1:** Demographic and clinical variables by conversion status.

	**Non-converters (*N* = 106)**	**Converters (*N* = 36)**	**Test statistic**	***p*-value**
Age at onset	21.38 (4.85)	19.63 (2.80)	***t*** **(117) = 1.876**	***p*** **= 0.042**
Age at assessment	29.17 (10.18)	29.39 (11.41)	*t*(142) = −0.108	*p* = 0.176
Sex (% female)	43.4%	30.1%	*X*^2^ (1) = 1.844	*p* = 0.174
Race (% Caucasian)	78.3%	69.5%	*X*^2^ (5) = 6.027	*p* = 0.304
Education (years)^∧^	15.11 (1.85)	14.39 (2.98)	*t*(142) = 1.715	*p* = 0.860
Comorbid SUD	60.4%	36.1%	***X***^**2**^ **(1) = 6.375**	***p*** **= 0.012**
Comorbid anxiety disorder	42.5%	30.6%	*X*2 (1) = 1.593	*p* = 0.207
MADRS	15.07 (9.65)	13.85 (11.60)	*t*(139) = 0.606	*p* = 0.213
YMRS	12.03 (12.93)	9.91 (10.04)	*t*(140) = 0.882	*p* = 0.113
PANSS Positive	14.86 (7.60)	15.20 (7.44)	*t*(140) = −0.232	*p* = 0.922
PANSS Negative	11.95 (5.26)	13.75 (5.34)	*t*(137) = −1.686	*p* = 0.924
PANSS General	29.57 (8.50)	29.09 (9.51)	*t*(139) = 0.281	*p* = 0.526

*SUD, Substance use disorder; MADRS, Montgomery-Asberg Depression Rating Scale; YMRS, Young Mania Rating Scale; PANSS, Positive and Negative Syndrome Scale*.

∧ *Education was collected using the SCID coding system. This ordinal system was converted to years of education for ease of interpretation using the following convention: less than high school (10 years), graduated high school (12 years), part college or graduated 2-year college (14 years), graduated 4-year college (16 years), part or complete graduate/professional school (18 years), unknown*.

*Bold values represent comparisons that reached statistical significance*.

### Predictors of Diagnostic Change

A logistic regression analysis was performed to investigate the relationship between conversion status (converter vs. non-converter) and presence or absence of a SUD, sex, age at onset, and diagnostic category at first assessment (mood disorder or SZ spectrum). The full model was significant [χ^2^(4) = 19.041, *p* = 0.001]. Presence or absence of a SUD was a significant predictor variable in the model (*p* = 0.007, OR = 3.621). Age at onset, sex, and diagnostic category were not significant independent predictors in the model.

## Discussion

This study was a retrospective analysis of the stability of SCID-IV-TR and SCID-5 diagnoses in 142 individuals with psychotic disorders enrolled in studies at McLean Hospital's Schizophrenia and Bipolar Disorder Program between 2006 and 2021. As hypothesized, we found that 25% of the overall sample experienced a change in diagnosis. Among the participants who underwent diagnostic change, diagnostic change from BP to SZA (81.8%) was more prevalent than the reverse (12.5%). Studies of diagnostic instability in psychotic disorders have produced inconsistent findings, with stability rates ranging from 0% to 96.5%, depending on sample type, sample size, stage of illness, and diagnosis. In our sample of 142 individuals, the overall stability rate was 74.65%, much higher than some stability rates in first-episode clinical samples (12, 15), but lower than those in some research samples (14). When broken down by diagnosis, the stability rates in our sample are generally reflective of previous findings. Specifically, stability rates of BP (86.9%), SZ (66.7%), Psych NOS (33.3%), and SZform (33.3%) in our sample are similar to most previous reports. SZA had a stability rate of 63.6% in our sample. As noted above, stability of the SZA classification has been highly variable across studies [1–6], which may reflect findings of (12, 13) shared and distinct phenomenological and genetic associations across the Kraeplinean divide (24).

Few baseline demographic or clinical variables differed by diagnostic conversion status. Age at onset and presence of a comorbid SUD differed by group, but only SUD remained a significant predictor in our multivariate regression model. Contrary to our hypotheses and previous findings, SUD was associated with non-conversion. One possibility is that in our sample SUDs occurred more frequently in people with BPI (63.1%) than other diagnoses [*X*^2^ (1, *N* = 142) = 6.375, *p* = 0.012] and the BPI group also showed greater diagnostic stability. These findings suggest that overall, demographic indicators and clinical presentation at the initial assessment may not serve as useful tools to predict whether or not patients will remain diagnostically stable over time.

Of note, while a quarter of our sample experienced a diagnostic switch, no participant moved outside of the psychosis spectrum with regard to primary diagnosis. These findings suggest that our tools for evaluation of broad diagnostic categories are reasonably reliable over time. At the same time, we must recognize the potential limitations of specific diagnostic labels and minimize the disadvantages of our current taxonomic systems by being open to reformulation over time (25, 26). As noted by Goldberg (27), diagnostic categories ignore real associations amongst diagnoses by drawing artificial boundaries between them. Use of diagnosis as a heuristic upon which to begin the processes of conceptualization rather than a static label designed to differentiate groups may actually help identify mechanisms related to the evolution of the syndrome over time. Such an approach may also facilitate identification of subsets of people who share common trajectories, which may benefit both clinical care and research (27). For example, we found varying rates of diagnostic stability by initial diagnosis in our sample, with the highest being in people initially diagnosed with BPI. Additionally, in people whose diagnoses changed over time SZA was the most common follow-up diagnosis to which people converted. Better understanding of the natural evolution of symptoms, such as indicated in Kraepelin's later proposed continuum of health to illness, and their relationship to stability or change may inform both treatment approaches and identification of biomarkers and common trajectories of illness.

In a research context, the current system of categorizing participants based on DSM or ICD diagnosis remains common, but may ultimately mis-classify a sizable minority of study subjects who would go on to a different diagnosis if assessed later, or artificially dichotomize people who share real phenomenological or biological overlap. The National Institute of Mental Health presents an alternative method for investigating mental illnesses and their impacts through a recent approach to diagnosis outlined in the Research Domain Criteria (RDoC). Insel and colleagues (28) assert that illnesses that may appear clinically different can result from the same etiology. Conversely, people classified as having the same disorder may show different patterns of illness course premorbidly and post-onset (29). Both phenomena dilute our ability to identify biomarkers of illness or link mechanisms to outcomes. Considering a combination of categorical, dimensional, and longitudinal factors may maximize our ability to identify critical biomarkers and mechanisms, and their dynamic interplay over time (30). Of course, the present findings cannot disentangle the source of diagnostic change, which could be attributed to a number of factors including measurement factors, participant reporting, or true change in symptomatology. It is possible that these illnesses are better conceptualized as syndromes, and that the related diagnostic categories under study here include symptom complexes that often occur in combination, leading to some fluidity of the placement of the “boundary” from one timepoint to another (27).

Several limitations should be noted, including relatively small sample size, some missing data due to this sample being drawn from several studies using different protocols, and inconsistent duration of time between assessments due to the nature of the retrospective analysis. The sample may also not be representative, as participants in these studies are people who self-select to participate in research studies at more than one timepoint. Additionally, all participants were between the ages of 18 and 60 limiting our ability to examine diagnostic stability in children and older adults. Replication of these findings in representative clinical populations (e.g., all consecutive inpatient admissions) are needed to address this issue. Although our sample size was similar to many other studies in the existing literature on diagnostic change, some findings that trended toward significance may have been limited by our sample size. In addition to sample restrictions, there were item-level dataset limitations that did not permit for full analyses of some potential predictors of diagnostic instability, such as auditory hallucinations. Because these data were aggregated from a number of different studies, the time interval between assessments and other methodological factors were not consistent. Prospective longitudinal assessment would address this issue, and permit examination of when in the course of illness diagnostic changes are likely to occur.

### Conclusion

Overall diagnostic changes occurred in 25% of our sample, although all participants' primary diagnoses remained within the psychosis spectrum. Among the participants who underwent diagnostic change, diagnostic change from BP to SZA (81.82%) was more prevalent than the reverse (12.50%). It was found that primary diagnosis (mood disorder or SZ spectrum disorder) at initial SCID and lack of SUDs were significantly associated with diagnostic conversion. Few studies report face-to-face diagnostic assessments using well-validated instruments at multiple timepoints. Additionally, we included a wide age range of participants permitting evaluation of age and duration of illness as factors associated with diagnostic change.

## Data Availability Statement

The data supporting the conclusions of this article will be made available upon request, as appropriate, without undue reservation.

## Ethics Statement

All procedures for the studies included herein were approved by the McLean Hospital Institutional Review Board or the Mass General Brigham Institutional Review Board. All participants provided written informed consent prior to participation.

## Author Contributions

AW and AC: conceptualization and design, drafting of manuscript, and interpretation of data. AW: acquisition and statistical analysis. DO, AS, and KL: critical revision of the manuscript. KL: supervision. All authors contributed to the article and approved the submitted version.

## Funding

This work was supported by the National Institute of Mental Health grants P50MH115846 (DO) and R01MH117012 (KL). Open access publication fees were funded by McLean Hospital's Schizophrenia and Bipolar Disorder Program.

## Conflict of Interest

The authors declare that the research was conducted in the absence of any commercial or financial relationships that could be construed as a potential conflict of interest.

## Publisher's Note

All claims expressed in this article are solely those of the authors and do not necessarily represent those of their affiliated organizations, or those of the publisher, the editors and the reviewers. Any product that may be evaluated in this article, or claim that may be made by its manufacturer, is not guaranteed or endorsed by the publisher.
